# The Spatiotemporal Variation and Ecological Evaluation of Macroinvertebrate Functional Feeding Groups in the Upper Yellow River

**DOI:** 10.3390/biology13100791

**Published:** 2024-10-02

**Authors:** Peilun Li, Shuhan Xiong, Jiacheng Liu, Tai Wang, Yanbin Liu, Kai Liu, Yongjie Wang, Jilong Wang

**Affiliations:** 1 Heilongjiang River Fisheries Research Institute, Chinese Academy of Fishery Sciences, Harbin 150070, China; lipeilun@hrfri.ac.cn (P.L.);; 2 Scientific Observing and Experimental Station of Fishery Resources and Environment in Heilongjiang River Basin, Ministry of Agriculture and Rural Affairs, Harbin 150070, China; 3 Gansu Fisheries Research Institute, Lanzhou 730030, China; 4 Ningxia Fisheries Research Institute, Yinchuan 750001, China

**Keywords:** Yellow River, macroinvertebrate, functional feeding groups, environmental factors

## Abstract

**Simple Summary:**

To elucidate the characteristics of functional feeding groups among macroinvertebrates during various months in the upper Yellow River, and their relationship with environmental factors, 33 sampling points were strategically placed across both gorge and plain regions. Collectively, these areas hosted 65 taxonomic units (genus or species) of macroinvertebrates, among which the collector-gatherers were predominantly dominant. Notably, *Gammarus* sp. and *Limnodrilus hoffmeisteri*, among the collector-gatherers, emerged as the prevalent species across both the gorge and plain areas. Mantel tests indicated that dissolved oxygen, conductivity, and orthophosphate were significant environmental determinants influencing the functional feeding groups of macroinvertebrates. Evaluations based on the Hilsenhoff Biological Index and the Shannon–Wiener Index suggest that the water quality of the upper Yellow River is moderate. An analysis using functional feeding group parameters showed a progressive increase in the biomass of macroinvertebrates from upstream (gorge areas) to downstream (plain areas), accompanied by enhanced habitat stability. Cascade hydropower development was pinpointed as a crucial factor impacting habitat stability. These insights provide relevant data and a theoretical basis for the conservation of aquatic biological resources and watershed management in the upper Yellow River.

**Abstract:**

Against the backdrop of hydropower development in the upper Yellow River, comprehending the spatiotemporal variation and ecological evaluation of macroinvertebrate functional feeding groups (FFGs) is paramount for the conservation and restoration of aquatic biological resources in watersheds. Detailed surveys of macroinvertebrates were conducted in the gorge and plain areas of the upper Yellow River in July 2022 and March, May, and October 2023, culminating in the identification of 65 taxonomic units (genus or species) spanning 4 phyla, 14 orders, and 35 families. Of these, 41 taxonomic units were discovered in the gorge areas and 57 in the plain areas. Among the FFGs of macroinvertebrates in the upper Yellow River, collector-gatherers were overwhelmingly dominant, followed by scrapers, collector-filterers, predators, and shredders. Concerning river section types, dominant species in the gorge areas included *Gammarus* sp., *Limnodrilus hoffmeisteri*, and *Polypedilum* sp. among collector-gatherers, while in the plain areas, dominant species included *Ecdyonurus* sp. among scrapers, *Hydropsyche* sp. among collector-filterers, and *Gammarus* sp., *Limnodrilus hoffmeisteri*, and *Chironomus* sp. among collector-gatherers. A Mantel test revealed that dissolved oxygen, conductivity, and orthophosphate were the primary environmental factors affecting the FFGs of macroinvertebrates in the upper Yellow River, with variations observed in their effects across different months. The evaluation results of the Hilsenhoff Biological Index and Shannon–Wiener Index indicate that the water quality of the upper Yellow River is at a moderate level. An assessment of the upper Yellow River ecosystem using FFG parameters demonstrated that macroinvertebrate biomass progressively increased from upstream (gorge areas) to downstream (plain areas) spatially, accompanied by increasing habitat stability, with cascade hydropower development identified as a key factor impacting habitat stability. These findings provide pertinent data and a theoretical foundation for the protection of aquatic biological resources and watershed management in the upper Yellow River.

## 1. Introduction

Macroinvertebrates serve as a natural food source for fish and play a crucial role in the decomposition of organic waste in aquatic environments [1]. As intermediaries in the food chain, they facilitate the breakdown of organic matter, the transformation of nutrients, and accelerate the self-purification process of rivers, making them an essential component of river ecosystems [2,3]. Moreover, macroinvertebrates are regarded as vital indicators for assessing the ecological status of aquatic ecosystems due to their species diversity, ease of collection, long life cycles, sensitivity to changes in water quality, and predictable changes in community structure following disturbances [4]. The functional feeding groups (FFGs) of macroinvertebrates refer to macroinvertebrate communities in river ecosystems with similar feeding habits. These groups primarily reflect the effects of habitat changes on macroinvertebrate communities based on the types of food resources available and the morphological adaptations associated with food acquisition, thereby revealing the structural characteristics of these communities and their adaptability to the habitat [5]. Based on their feeding habits, they are categorized into five FFGs: collector-filterers, collector-gatherers, predators, scrapers, and shredders [6,7].

The morphology and structural characteristics of rivers are influenced by changes in regional climate and geographical features [8]. As the seasons change, both the climate and geography of the watershed will consequently change. The alterations in river flow, water temperature, and the accumulation of fallen leaves between different seasons all impact the FFGs of macroinvertebrates [5,9]. The alternation of seasons is a significant factor influencing the temporal variation in the FFGs of macroinvertebrates. In order to adapt to the seasonal changes in the river environment, macroinvertebrates often adopt different life history strategies and appear in a certain order on the timeline to occupy different ecological niches at different time scales [10]. For example, during periods of high water levels and frequent floods in the wet season, macroinvertebrates will build or seek “refuges” to evade the dangers brought by floods [11]. Studying the seasonal dynamic changes in macroinvertebrates can provide a better understanding of the periodic circulation of river biota, the dynamics of community migration, and their ecological effects [3,12]. Therefore, it is of great significance to investigate the spatiotemporal variation characteristics of the FFGs of macroinvertebrates and establish the response relationship between the FFGs and the environmental factors to effectively indicate the health status of the river ecosystem and the ecological restoration of the river [5,6].

The Qinghai–Tibet Plateau is noted for having an extremely delicate ecosystem, and its water ecosystems are even more susceptible to environmental stress [13]. The Yellow River, originating on the Qinghai–Tibet Plateau and coursing through nine provinces and autonomous regions, emerges as the second largest river in China, and is distinguished globally for its substantial sediment load. It spans a total length of 5464 km and encompasses a drainage area of 752,443 km^2^ [13,14]. The upper reaches of the Yellow River feature a wide range of latitudes, significant changes in elevation, and diverse climates and topographies. As a result, its aquatic ecosystem is extremely sensitive and fragile, making it a focal point for biodiversity research in river networks. Based on different fluvial characteristics, the upper part of the Yellow River can be segmented into three sectors: the headwaters, the gorge area, and the plain area [15]. Nonetheless, the aquatic ecosystem and biodiversity in this part of the Yellow River are under serious threat due to the socio-economic developments and subsequent direct and indirect pressures induced by human activities. These activities include, but are not limited to, the execution of hydraulic engineering projects, alterations in land usage patterns, proliferation of invasive alien species, and pollution affecting water quality. This scenario is exemplified by the 21 hydropower stations erected in the gorge area of the upper Yellow River, causing severe fragmentation of river habitats, subsequently altering both the community structure and diversity of aquatic organisms within the area [16,17]. Consequently, the aquatic environment of the upper Yellow River has consistently been the locus of scientific scrutiny, with specific emphasis directed towards its ecology and biodiversity [13,14,15,16,17].

Currently, foreign research predominantly emphasizes the establishment and utilization of assessment systems based on the FFGs of macroinvertebrates, as well as the response mechanism of such groups towards human endeavors [18,19]. While in contrast, in China, there exists a discernible scarcity of comprehensive analysis of the effects of temporal and spatial fluctuations in river habitat conditions on the FFGs of macroinvertebrates. Moreover, understanding the structural nuances of these groups plays an indispensable role in evaluating the overarching spatiotemporal condition of river basin habitats [20]. In addition, there is a palpable dearth of systematic research on macroinvertebrates inhabiting the upper sections of the Yellow River, which has resulted in an incomplete understanding of their community structures and the factors influencing them. Therefore, the current research highlights an extensive study of the stretch of the Yellow River from Longyangxia to Shizuishan section. Utilizing data from sampling surveys conducted between 2022 and 2023, this study aspires to scrutinize the spatiotemporal variations in the FFGs of macroinvertebrates to decipher the relationship between these groups and the associated environmental factors in the river. It further aims to assess the ecological parameters based on FFGs with a view to evaluating the health status of the ecosystem in the upper Yellow River, thereby providing a theoretical premise for biological assessment of water quality and ecological restoration efforts targeted at the river.

## 2. Material and Methods

### 2.1. Study Area

**Figure 1:**
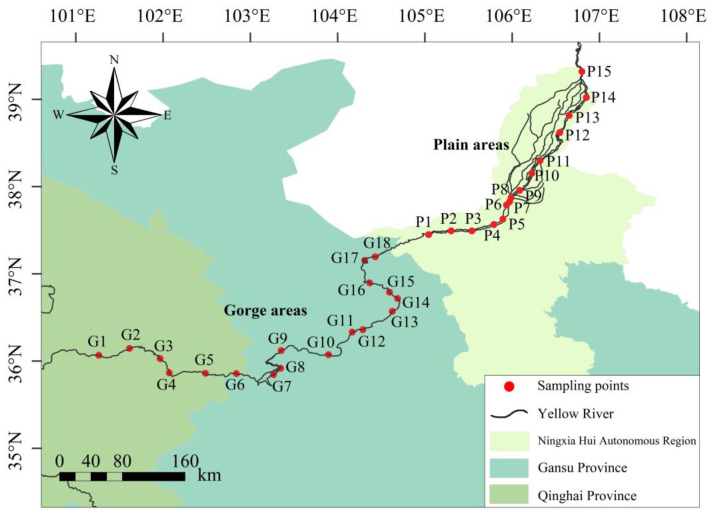
Diagram of sampling points in the gorge and plain areas of the upper Yellow River (the latitude and longitude of sampling points are shown in Table S1).

The section of the Yellow River’s mainstream extending from Longyangxia to Shizuishan, located in the upper Yellow River, spans approximately 1100 km and experiences an aggregate drop of a similar magnitude of around 1100 m. The segment that stretches from Longyangxia in Qinghai Province to the Xiaheyan section in Ningxia Hui Autonomous Region is characterized as a gorge area. This particular area, a substantial reservoir of Yellow River’s hydropower resources, hosts a conglomerate of mainstream cascade hydropower stations, with 21 such entities currently in operation [16]. The section transitioning from Xiaheyan to Shizuishan in Ningxia Hui Autonomous Region unveils vast alluvial plains. The surrounding terrain predominantly consists of desert and desert grasslands, accompanied by gentle riverbeds and tranquil water flow. Expansive alluvial plains encompass both sides of the river banks, bestowing upon it a renowned reputation for the diversion of water from the Yellow River to fulfill irrigation purposes. Predicated upon the hydrological conditions and geomorphological characteristics of the Yellow River’s upper reaches, 33 sampling points were demarcated (Figure 1). Among them, 18 sampling points were established between the Longyangxia and Xiaheyan sections, while the remaining 15 were situated within the stretch from Xiaheyan to the Shizuishan section (Table S1). Each of these sampling points served as the center for macroinvertebrate collection and determination of water environmental factors, carried out in July 2022 and in March, May, and October 2023, respectively.

### 2.2. Investigation of Macroinvertebrates

Following the guidelines for “Biodiversity Observation Techniques for Freshwater Benthic Macroinvertebrates (HJ710.8-2014)” [21], D-shaped nets (with a mouth diameter of 30 cm and mesh size of 500 μm) and Peterson grab samplers (with an opening area of 1/16 m^2^) were employed to collect macroinvertebrate samples from the Yellow River’s mainstream. Each sampling point encompassed a 100 m river segment based on habitat characteristics. Sampling was repeated 5 to 10 times at each point and combined into a single sample. On-site, samples were sieved and washed with a 40-mesh sieve, after which macroinvertebrates were individually selected, fixed in a 4% formalin solution by volume, labeled, and preserved. The samples were then transported to the laboratory for identification to the lowest possible taxonomic level, typically genus or species [22,23,24]. Finally, macroinvertebrates from each sampling point were counted, weighed by species, and converted into density (ind./m^2^) and biomass (g/m^2^) based on the sampling area.

### 2.3. Measurement of Environmental Factors

During the survey, a YSI multiparameter water quality monitoring analyzer (YSI ProQuatro, Dayton, OH, USA) was employed to measure water temperature (T, °C), pH, dissolved oxygen (DO, mg/L), and conductivity (Cond, μS/cm) at each sampling point. Concurrently, 1 L water samples were collected at each location, refrigerated, and transported to the laboratory. Upon arrival, following the “Methods for the Monitoring and Analysis of Water and Wastewater (4th edition)” [25], orthophosphate (PO_4_^3−^, mg/L), total phosphorus (TP, mg/L), total nitrogen (TN, mg/L), ammonia nitrogen (NH_3_-N, mg/L), chemical oxygen demand (CODMn, mg/L), chlorophyll-a (Chl-a, μg/L), suspended solids (SSol, mg/L), and suspended sediment (SSed, mg/L) were analyzed.

### 2.4. Calculation of Dominance Index

The species dominance index (*Y*) is used to indicate the degree of dominance of a species within a functional feeding group of macroinvertebrates. The formula is as follows:

$$Y=\left(n_{i}/N\right)f_{i}$$

In the equation, *N* represents the total density of large benthic animals within each functional feeding group (ind./m^2^), *n_i_* is the density of the ith species (ind./m^2^), and *f_i_* is the frequency of occurrence of the *i*th species. Species with a dominance index *Y* > 0.02 are identified as dominant species within each functional feeding group surveyed in this study [26].

### 2.5. Classification of Macroinvertebrate FFGs and Delineation of Ecosystem Attributes

Macroinvertebrates in the upper Yellow River are categorized into five FFGs: collector-gatherers, predators, scrapers, collector-filterers, and shredders [7,27]. Drawing on the research by Yoshimura et al. [28] and Zhang et al. [29], this study evaluates the ecosystem of the upper Yellow River using parameters of macroinvertebrate FFGs. These parameters encompass material cycling (F1–F4), longitudinal transport (F5, F6), lateral input (F7, F8), and other factors (F9–F11), totaling 11 parameters (Table 1).

### 2.6. Biological Evaluation of Water Quality

The assessment of water quality is conducted using the Hilsenhoff Biological Index (*HBI*) and the Shannon–Wiener Index (*H*′), with the formulas for calculation as follows:

$$HBI=\sum_{i=1}^{n}\frac{n_{i}\cdot X_{i}}{N}$$

$$H^{\prime }=\sum_{i=1}^{S}P_{i}\log_{2}P_{i}$$

In the formula, *N* is the total number of individuals; *n_i_* is the number of individuals in the *i*-th taxonomic unit (genus or species); *X_i_* denotes the pollution tolerance value of the *i*-th taxonomic unit (genus or species); *S* represents the total number of species; and $P_{i}=n_{i}/N$. 

The pollution tolerance values for macroinvertebrates refer to the verified values in the “Technical Guide for Aquatic Ecological Monitoring and Evaluation: River Aquatic Biological Monitoring and Evaluation (Trial)” (HJ 1295-2023) [30]. For species not listed in the guide, the pollution tolerance values are determined according to the classification by Wang and Yang (2004) [31]. In this study, the Hilsenhoff Biological Index is categorized into five levels: <3.9 (Excellent), 3.9–5.4 (Good), 5.4–7.0 (Moderate), 7.0–8.5 (Bad), and >8.5 (Very bad) [30]. The Shannon–Wiener Index is divided into five classes: >3.0 (Excellent), 2.0–3.0 (Good), 1.0–2.0 (Moderate), 0–1.0 (Bad), and =0 (Very bad) [30].

### 2.7. Statistical Analysis

The original data were compiled and processed using Excel 2017. Disparities in the density of FFGs among macroinvertebrates were assessed with the Mann–Whitney U test in SPSS 22.0 (SPSS for Windows, IBM, Armonk, NY, USA). Histograms were generated using GraphPad Prism 8 (Graphpad, San Diego, CA, USA). The PERMANOVA test, facilitated by the Vegan package in R 4.3.1, examined the variations in spatial distribution among the FFGs of macroinvertebrates. Simultaneously, the Mantel test explored correlations between macroinvertebrate FFGs and environmental factors in the upper Yellow River.

## 3. Results

### 3.1. Community Structure of Macroinvertebrate FFGs

Throughout the investigation period, a total of 65 taxonomic units (genus or species) belonging to 4 phyla, 14 orders, and 35 families of macroinvertebrates were identified from the Longyangxia section to the Shizuishan section in the upper Yellow River (Table S2). Among these, predator species constituted the highest number, totaling 23 species and representing 35.38% of the overall species count. Following closely were collector-gatherer species, numbering 16 and constituting 24.62% of the total species. Scraper species accounted for 13 species, making up 20.00% of the total, and shredder species accounted for 9 species, representing 13.85% of the total species. Conversely, collector-filterer species were the least numerous, comprising only four species, which equated to 6.15% of the total. Regarding the segmentation of river types, 41 taxonomic units of macroinvertebrates were identified in the gorge area, while 57 taxonomic units were recorded in the plain area (Table S2). The density of macroinvertebrate FFGs in different months and river sections in the upper Yellow River was analyzed using a PERMANOVA test (Tables S3–S6). The results indicated significant differences in the density of macroinvertebrate FFGs among different months (*R*^2^ = 0.086, *F* = 4.697, *p* = 0.001) and among different river sections (*R*^2^ = 0.126, *F* = 20.623, *p* = 0.001) (Table 2).

Upon examining the selected months, 12 dominant species spanning five FFGs were revealed in the upper Yellow River (Table 3). Among these, *Ecdyonurus* sp. from the scrapers, as well as *Chironomus* sp., *Polypedilum* sp., *Limnodrilus hoffmeisteri*, and *Gammarus* sp. from the collector-gatherers, emerged as commonly dominant species in both the gorge and plain areas. Furthermore, *Palaemon modestus* and *Palaemonetes sinensis* from the shredders, *Limnodrilus* sp. from the collector-gatherers, *Cryptochironomus* sp. from predators, and *Hydropsyche* sp. from the shredders were dominant species exclusive to the plain area. Conversely, *Baetis* sp. and *Tubifex sinicus* from the collector-gatherers were species predominantly found in the gorge area. With regard to river section types, dominant species in the gorge area were primarily classify as collector-gatherers, with *Gammarus* sp. exhibiting the highest dominance value at 0.225, followed by *Limnodrilus hoffmeisteri* (0.095), and *Polypedilum* sp. (0.064). In the plain area, the dominant species primarily include *Ecdyonurus* sp. from scrapers (0.091), *Hydropsyche* sp. from collector-filterers (0.087), as well as *Gammarus* sp. (0.048), *Limnodrilus hoffmeisteri* (0.042), and *Chironomus* sp. (0.021) from collector-gatherers.

### 3.2. Spatiotemporal Variation Characteristics of Macroinvertebrate FFGs

**Figure 2:**
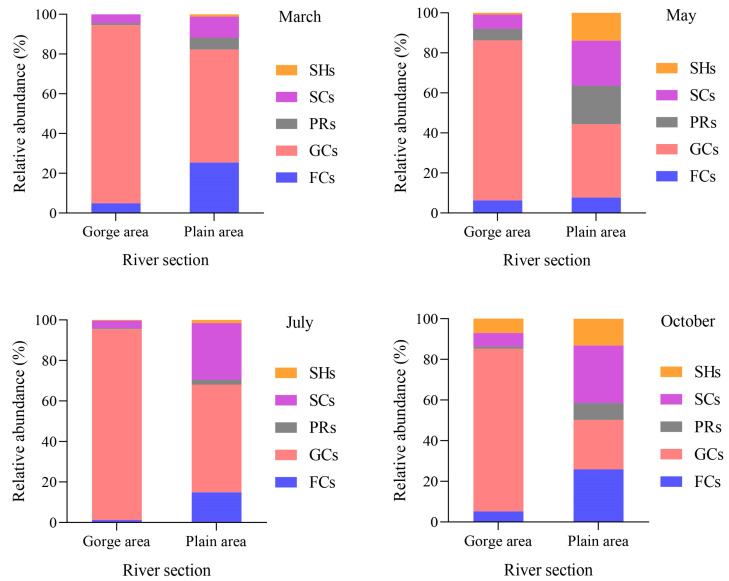
The relative abundance of macroinvertebrate FFGs in the upper Yellow River among different river sections in different months. Note: CGs = collector-gatherers; PRs = predators; SCs = scrapers; CFs = collector-filterers; SHs = shredders.

The distribution of the FFGs of macroinvertebrates’ relative abundance in the upper Yellow River is illustrated in Figure 2. Among these groups, collector-gatherers consistently exhibit higher relative abundance across all river segments and months, underscoring their dominance. In March, the relative abundance of collector-gatherers reached its zenith in the gorge area, soaring to 89.70%, while October marked its nadir in the plain area, registering at 24.35%. Conversely, scrapers peaked in relative abundance in the plain area in October at 28.34%, contrasting with their lowest point in July within the gorge area, at 3.75%. Similarly, collector-filterers exhibited their maximum relative abundance in the plain area during October, reaching 25.86%, and their minimum in the gorge area in July, at 1.21%. Predators peaked in the plain area in May at 19.20%, and reached their lowest point in the gorge area in July at 0.37%. As for shredders, their highest relative abundance was recorded in the plain area during May, peaking at 13.88%, while the lowest was observed in the gorge area in March, where this functional group was not collected.

**Figure 3:**
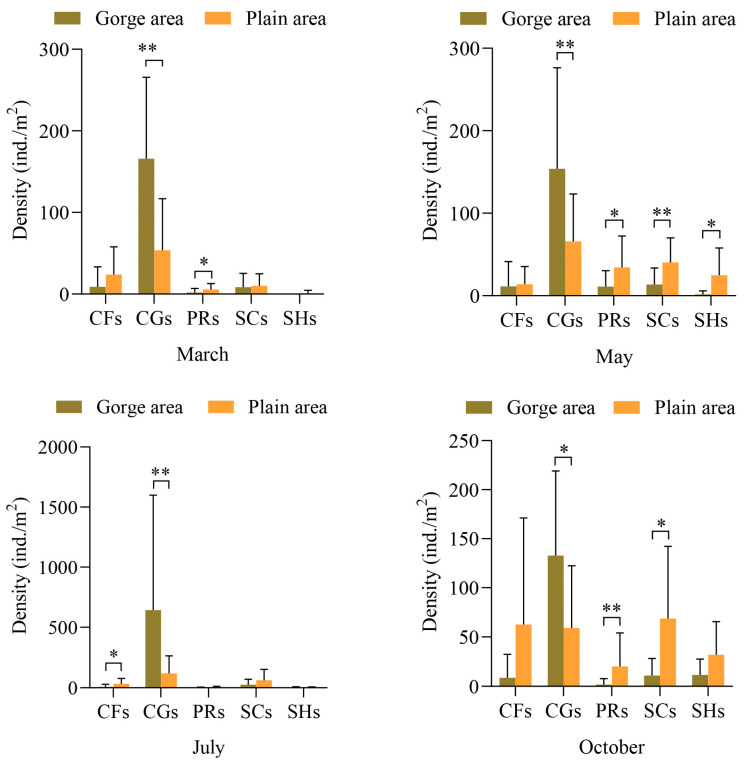
Density of macroinvertebrate FFGs in the gorge and plain areas in different months. Note: CGs = collector-gatherers; PRs = predators; SCs = scrapers; CFs = collector-filterers; SHs = shredders. “*” represents significant difference (*p* < 0.05), and “**” represents extremely significant difference (*p* < 0.01).

The distribution of macroinvertebrate FFG densities in the upper Yellow River is depicted in Figure 3. The Mann–Whitney U test reveals that the average density of collector-filterers in the plain area consistently surpasses that in the gorge area, with highly significant differences observed between the two river sections in July (*Z* = −2.271, *p* = 0.023), while no significant differences were noted in other months. Conversely, the average density of collector-gatherers in the plain area is lower than in the gorge area across various months, with significant differences in October (*Z* = −2.498, *p* = 0.012) and very significant differences in March (*Z* = −2.498, *p* = 0.012), May (*Z* = −2.623, *p* = 0.009), and July (*Z* = −3.725, *p* = 0.000). The average density of predators in the plain area is higher than in the gorge area, with notably significant differences in March (*Z* = −2.160, *p* = 0.031) and May (*Z* = −1.978, *p* = 0.048), and very significant differences in October (*Z* = −3.041, *p* = 0.002). Similarly, the average density of scrapers in the plain area is higher than in the gorge area across different months, with very significant differences observed in May (*Z* = −2.420, *p* = 0.016), significant differences in October (*Z* = −2.650, *p* = 0.008), and no differences between March and July. Additionally, the average density of shredders in the plain area is higher than in the gorge area across different months, with significant differences observed between river sections in May (*Z* = −2.586, *p* = 0.010), while no differences were noted in other months.

### 3.3. Relationships between Macroinvertebrate FFGs and Environmental Factors

**Figure 4:**
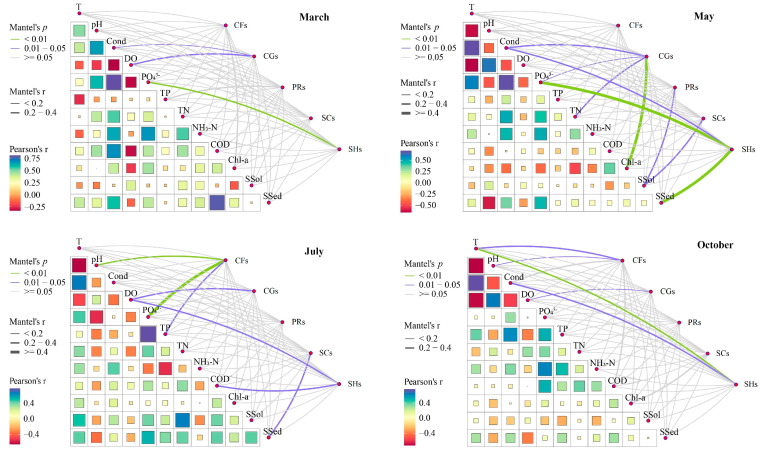
Relationships between macroinvertebrate FFGs and environmental factors obtained by Mantel test in different months in the upper Yellow River. Note: CGs = collector-gatherers; PRs = predators; SCs = scrapers; CFs = collector-filterers; SHs = shredders; T = water temperature; DO = dissolved oxygen; Cond = conductivity; PO_4_^3−^ = orthophosphate; TP = total phosphorus; TN = total nitrogen; NH_3_-N = ammonia nitrogen; CODMn = chemical oxygen demand; Chl-a = chlorophyll-a; SSol = suspended solids; SSed = suspended sediment.

A meticulous examination reveals that substantial correlations exist between environmental factors (Table S7) and the macroinvertebrate FFGs in the upper reaches of the Yellow River, as evidenced by the Mantel test (Figure 4). In March, the density of collector-gatherers among the macroinvertebrates displayed significant correlations with Cond (*p* = 0.013) and DO (*p* = 0.023). Similarly, the density of shredders was significantly correlated with PO_4_^3−^ (*p* = 0.006). In May, the collector-gatherer density showed significant correlations with Chl-a (*p* = 0.003), as well as Cond (*p* = 0.025), TN (*p* = 0.011), and PO_4_^3−^ (*p* = 0.037). The densities of predators and scrapers were significantly correlated with SSol (predators: *p* = 0.046; scrapers: *p* = 0.049). The shredder density was significantly correlated with PO_4_^3−^ (*p* = 0.001), SSel (*p* = 0.001), and Cond (*p* = 0.018). In July, the collector-filterer density demonstrated significant correlations with PO_4_^3−^ (*p* = 0.004) and pH (*p* = 0.009), as well as TP (*p* = 0.039). The collector-gatherer density was significantly correlated with DO (*p* = 0.048), while the scraper density exhibited a significant correlation with SSel (*p* = 0.047). The shredder density showed significant correlations with DO (*p* = 0.018) and CODMn (*p* = 0.029). By October, the collector-filterer density displayed significant correlations with T (*p* = 0.047) and pH (*p* = 0.020), while the collector-gatherer density was significantly correlated with DO (*p* = 0.039). Additionally, the density of shredders showed significant correlations with T (*p* = 0.009) and Cond (*p* = 0.024). 

### 3.4. Ecological Assessment of Macroinvertebrate FFGs Ecosystem

Based on the parameters of FFGs, the results of a spatiotemporal difference analysis are shown in Table 4. The macroinvertebrate material cycle is characterized by a gradual increase in primary production (F1), secondary production (F2), and autotrophy/heterotrophy (F3) from the gorge area to the plain area; in contrast, decomposition (F4) shows a trend toward higher levels in the gorge area relative to the plain area. According to a month-by-month analysis, F1 and F4 peak in July, F2 and F3 peak in October, and all four parameters (F1, F2, F3, and F4) reach their lowest points in March. In terms of longitudinal transport, there is a steady climb from the gorge area to the plain area in both longitudinal transport (F5) and relative longitudinal transport (F6). Regarding lateral input, with peaks in October and lows in March, the plain area has higher lateral input (F7) and relative lateral input (F8) than the gorge area. Other aspects of the plain region relative to the canyon area are higher, though often at lower parameter levels: the CPOM input/FPOM input (F9), top-down predator management (F10), and habitat stability (F11). In a range of months, F9 reaches its maximum in October, F10 reaches its maximum in May, and F11 reaches its maximum in July.

An assessment of the ecosystems in the gorge and plain areas of the upper Yellow River, based on the thorough analysis above, indicates that macroinvertebrate biomass progressively rises spatially from upstream (gorge area) to downstream (plain area), accompanied by an increase in habitat stability. The upper Yellow River ecosystem shows greater parameter levels temporally between July and October, suggesting enhanced habitat stability during these months. It is important to note, nonetheless, that the overall level of parameter F10 is low, indicating that the upper Yellow River ecosystem is weakly regulated by high trophic levels of macroinvertebrate species on lower trophic levels [32].

### 3.5. The Biological Evaluation of Water Quality

The evaluation results of the Hilsenhoff Biological Index (*HBI*) and Shannon–Wiener Index (*H*′) are used for water quality analysis (Table 5). The analysis of the *HBI* reveals fluctuations in water quality at various time points throughout the year. Across different river sections, the gorge areas displayed good water quality in March and regular moderate levels thereafter, whereas plains areas were consistently closer to a moderate level, with a notable improvement observed in July. In parallel, the *H*′ reflected a generally analogous trend, mapping the oscillations in water quality induced by cyclic changes. Throughout different segments, the water quality remained moderate, albeit with a gentle inclination towards good conditions in the plain areas during May. Ultimately, both the *HBI* and *H*′ indices consistently denote a moderate water cleanliness across the upper reaches of the Yellow River, with slightly more favorable conditions in the plain areas compared to the gorge areas.

## 4. Discussion

In the upper Yellow River, the collector-gatherers dominate among macroinvertebrate FFGs, exhibiting higher densities compared to other groups. This finding is consistent with results from the Huangshui River [33] and the Xiangxi River [34], but contrasts with the Buyuan River, where collector-filterers are most abundant [4], the Lijiang River, where scrapers prevail [26], and the Qiaobian River, where collector-filterers are predominant [20]. The density of shredders is lowest in the upper Yellow River, similarly to the Buyuan River [4], the Lijiang River [26], and the Huangshui River [33], but differs from the Qiaobian River [20], where collector-gatherers are less favored. This comparison reveals both similarities and differences in the dominant and least favored FFGs of macroinvertebrates across various rivers. The primary factors contributing to these variations likely include the differing geographical scales of each river, distinct habitat conditions, and the species composition and distribution of macroinvertebrate FFGs, which reflect comprehensive responses to varied habitat factors [29,35].

Vannote et al. [36] proposed the River Continuum Concept (RCC), based on studies of undisturbed river ecosystems in North American temperate forests. According to RCC predictions, the relative abundance of collector-gatherers and collector-filterers gradually increases from upstream to downstream, while the relative abundance of shredders decreases along the same gradient [20,26,36]. Collector-gatherers and collector-filterers thrive in river ecosystems due to the concentration of organic matter in the water, which facilitates their efficient utilization and thereby stimulates their growth and reproductive rate [37]. In this study, the trend in the relative abundance of collector-filterers aligns with RCC predictions, whereas the relative abundance of collector-gatherers gradually decreases from the gorge area to the plain area, and the relative abundance of shredders increases along the same gradient, contrary to RCC predictions. Kerakova et al. [38] noted that the spatial distribution of macroinvertebrate FFGs is influenced by the heterogeneity of riverbed substrates, other environmental factors, and human activities. The relative abundance of collector-gatherers in the upper Yellow River gradually decreases from the gorge area to the plain area. Zhang et al. [20] identified that the natural sedimentation of organic debris in rivers is the main food source for collector-gatherers. The construction of cascade hydropower stations in the gorge area disrupts the ecological connectivity and longitudinal coherence of the river, transforming various rapids habitats into cascade reservoir habitats consisting of stagnant water zones, transition zones, and river zones [39]. The slower flow velocity favors the deposition of organic debris and particles, providing rich food for dominant species such as Gammarus sp. among the collector-gatherers. Furthermore, pollutants such as domestic sewage from coastal villages in the gorge area, manure from livestock farming, and residues of pesticides and fertilizers from agriculture are directly or indirectly discharged into the river, causing water pollution and increasing the dominance index of pollutant-tolerant populations such as *Polypedilum* sp. and *Limnodrilus hoffmeisteri* among the collector-gatherers. Additionally, shredders are considered as important as collector-gatherers in river headwaters in the context of RCC, as they utilize leaf litter and associated biomass to produce fine organic particles [40]. Shredders primarily feed on coarse organic matter, and changes in riparian vegetation can affect habitat characteristics, detritus quality, and invertebrate colonization rate (i.e., migration or dispersal) [41,42,43]. In this study, the relative abundance of shredders in the upper Yellow River gradually increases from the gorge area to the plain area. This could be due to the sparse vegetation on both sides of the mountains in the gorge area, leading to an insufficient input of organic waste such as branches and leaves, thus resulting in a lack of food for shredders in the gorge area [44]. Jiang et al. [34] found that the relative abundance of predators is mainly influenced by the water environment and flow velocity. In this study, the relative abundance of predators is higher in the plain area than in the gorge area, possibly due to the less disturbed riverbed, wider channels, and slower flow velocity in the plains, which are favorable for predatory groups such as Odonata and Coleoptera. Similarly, the relative abundance of scrapers is lower in the gorge than in the plain. This could be because the main food source for scrapers is periphyton, and the deeper water in the gorge sections impairs periphyton photosynthesis, leading to a lack of food resources for scrapers [4,36].

Environmental factors are pivotal components of river ecosystems that directly or indirectly influence community structure and the spatial and temporal distribution of aquatic organisms [18,26,29]. Identifying the key environmental factors impacting macroinvertebrate FFGs is essential for assessing the health of river ecosystems [45]. Studies have demonstrated that the effects of environmental factors on macroinvertebrates vary across different regions and months, adding complexity to their analysis [34,45]. In our study, Mantel test results indicate significant monthly variations in environmental factors affecting macroinvertebrate FFGs in the upper Yellow River. The densities of shredders in March, collector-gatherers in May, shredders in May, and collector-filterers in July all show a significant positive correlation with orthophosphate levels. Additionally, the density of collector-filterers in July is significantly positively correlated with total phosphorus. Previous research has indicated that elevated phosphorus concentrations in rivers lead to eutrophication, promoting the proliferation and breeding of collector-filterers within macroinvertebrate FFGs, thus favoring pollution-tolerant species [46,47]. In this study, *Chironomus* sp. and *Limnodrilus hoffmeisteri*, known pollution-tolerant species, are identified as collector-gatherers in the survey region. Wang et al. [48] highlighted that adequate dissolved oxygen content in water supports the feeding, growth, and reproduction of macroinvertebrate FFGs. Our study shows that the densities of collector-gatherers in March, July, and October are significantly correlated with dissolved oxygen, suggesting that higher dissolved oxygen levels within an appropriate range can enhance the feeding and growth of collector-gatherers such as *Gammarus* sp. Conductivity, a parameter measuring total dissolved ion content in water, primarily affects macroinvertebrates by influencing the osmotic pressure balance of cell membranes [49,50]. An increase in conductivity leads to a decline in sensitive species and a rise in pollution-tolerant species among macroinvertebrates [49]. Our findings reveal significant correlations between the densities of collector-gatherers in March and May and shredders in October with conductivity, while no significant correlation is observed between FFGs and conductivity in July. This is attributed to the higher conductivity in July compared to other months, with predominant species such as *Tubifex sinicus* and *Limnodrilus* sp. being pollution-tolerant, corroborating the results of Zhang et al. [49]. Moreover, the suspended solids content is significantly correlated with the densities of predators and shredders in May, while the suspended sediment content shows significant correlations with the densities of shredders in May and predators in July. This indicates that the influx of suspended solids and sediments during the flood period in the upper Yellow River affects macroinvertebrate FFGs [15]. Overall, dissolved oxygen, conductivity, and orthophosphate emerge as the main environmental factors influencing macroinvertebrate FFGs in the upper Yellow River, with notable variations in their effects on each FFG across different months.

Material cycling is one of the most crucial functions of ecological systems involving macroinvertebrates. The density of shredders reflects the material uptake capacity along riverbanks, while the density of collector-filterers embodies the uptake capacity of rivers longitudinally [27,47]. In certain sections of the upper Yellow River, the absence of shredders and collector-filterers indicates impaired longitudinal material input capacity and ecosystem connectivity. Compared to other Chinese rivers, the upper Yellow River’s macroinvertebrate FFGs exhibit significantly lower material cycling and longitudinal input capacity than those in inland rivers like the Lijiang River [26], the Yongding River Basin [47], and the Xinyang section of the main stream of the Huaihe River [29]. A spatial analysis of various FFG parameters reveals that decomposition (F4) in the gorge area is significantly higher than in the plain area. This is attributed to the higher densities of collectors and shredders in the gorge area. Consequently, the FFGs in the gorge area possess a greater decomposition capability than those in the plain area. Conversely, other FFG parameters are higher in the plain area, indicating better aquatic ecological health there. This disparity might stem from the sparse vegetation along the gorge’s banks, leading to lower material input and nutrient-poorer waters. Additionally, cascade dams in the gorge area prolong hydraulic retention time, causing nutrient and organic debris accumulation, which results in a notable retention effect [51,52]. In contrast, the plain area’s water body has a better flow and exchange capacity, enhancing the river’s longitudinal transport capacity and providing greater habitat stability [53]. Moreover, the general community parameters of different FFGs are higher in July and October than in March and May. Wang et al. [48] noted that within suitable temperature ranges, higher water temperatures promote feeding, reproduction, and other life activities of macroinvertebrates. Therefore, lower water temperatures and nutrient scarcity in March and May weaken the metabolism of FFGs, limiting biological survival and reducing FFG parameters [54]. Thus, the upper Yellow River is influenced by climatic characteristics and human activities, forming unique regional habitat features. Due to factors like sparse riparian vegetation and high habitat fragmentation, the habitat stability in the gorge area is lower than that in the plain area. 

Prior studies indicate that river water quality is generally superior upstream compared to downstream [55]. However, this research reveals that the water quality from Longyangxia to Shizuishan sections in the upper Yellow River is only at a moderate level, while the downstream plain areas exhibit slightly better water quality than the upstream gorge areas. This phenomenon may be attributed to severe industrial pollution in the gorge regions and the dense distribution of cascading hydropower stations, along with significant river fragmentation, which consequentially reduces the volume of water exchange in the river [56]. The analytical results illustrate disparities in the assessment of water quality at different sampling points using the Hilsenhoff Biological Index and the Shannon–Wiener Index. These differences arise primarily due to the distinct methodologies employed by the indices: the Shannon–Wiener Index evaluates water quality by analyzing the structure of the macroinvertebrate communities, without considering the species’ pollution tolerance levels; conversely, the Hilsenhoff Biological Index focuses primarily on the pollution tolerance of the species [57]. Consequently, biological evaluations of water quality should utilize appropriate indicators to carry out a comprehensive analysis that facilitates a complementary relationship among various parameters. Given that the upper Yellow River is a focal point for hydropower development, which significantly impacts the structure and distribution of benthic communities. Hence, it is crucial for the future restoration and protection of the upper Yellow River ecosystem to moderately restore and maintain flow in the gorge areas to ensure the river’s longitudinal connectivity.

## 5. Conclusions

In the upper reaches of the Yellow River, collector-gatherers predominate among the macroinvertebrate FFGs, while shredders find themselves at a disadvantage. An analysis of the spatiotemporal variation characteristics of macroinvertebrate FFGs revealed that the distribution of each FFG may mirror the temporal and spatial alterations in the habitat of the upper Yellow River, in addition to reflecting the impact of anthropogenic activities on the environment. These distribution patterns are notably influenced by spatiotemporal fluctuations in water and food availability resulting from hydropower development. Dissolved oxygen, conductivity, and orthophosphate constitute the principal environmental factors influencing macroinvertebrate FFGs in the upper Yellow River, exhibiting varied effects across different FFGs and throughout various months. The evaluation results of the Hilsenhoff Biological Index and Shannon–Wiener Index indicate that the water quality of the upper Yellow River is at a moderate level. An assessment of the ecosystem of the upper Yellow River using FFG parameters indicated that the habitat stability is significantly greater in plain areas than in gorge regions, with cascade hydropower development identified as a crucial factor impacting habitat stability.

## Figures and Tables

**Table 1:** FFGs of macroinvertebrates related to ecosystem attributes.

Item	Attributes Code	Ecosystem Attribute	Metrics Based on FFGs
Material cycling	F1	Primary production	Density of SCs
	F2	Secondary production	Biomass
	F3	Autotrophy/heterotrophy	Ratio of SCs to CFs and CGs
	F4	Decomposition	Density of SHs and CGs
Longitudinal transport	F5	Longitudinal transport	Density of CFs
	F6	Relative longitudinal transport	Ratio of CFs to SHs and CGs
Lateral input	F7	Lateral input	Density of SHs
	F8	Relative lateral input	Ratio of SHs to total density
Others	F9	CPOM input/FPOM input	Ratio of SHs to CFs and CGs
	F10	Top-down predator control	Ratio of PRs to total density
	F11	Habitat stability	Ratio of SCs and CFs to total SHs and CGs

Note: CGs = collector-gatherers; PRs = predators; SCs = scrapers; CFs = collector-filterers; SHs = shredders; CPOM = coarse particulate organic matter; FPOM = fine particulate organic matter. These abbreviations are the same in the following Figures and Tables.

**Table 2:** PERMANOVA test results of FFGs density spatial structure of macroinvertebrates in the upper Yellow River.

Groups	*df*	Sum of Squares	*R*-Squared	*F* Statistics	*p*-Value
River section	1	3.726	0.126	20.623	0.001
Month	3	2.546	0.086	4.697	0.001
River section × Month	3	0.914	0.031	1.687	0.064
Residual	124	22.406	0.757		
Total	131	29.593	1.000		

**Table 3:** Dominant species and FFGs of macroinvertebrates in the upper Yellow River.

Dominant Species	FFGs	Gorge Area					Plain Area				
		March	May	July	October	Total	March	May	July	October	Total
*Palaemon modestus*	SHs									0.073	
*Palaemonetes sinensis*	SHs							0.039			
*Ecdyonurus* sp.	SCs			0.034			0.028	0.088	0.113	0.104	0.091
*Cryptochironomus* sp.	PRs							0.058			
*Baetis* sp.	CGs				0.046						
*Chironomus* sp.	CGs		0.030		0.052		0.027	0.101			0.021
*Polypedilum* sp.	CGs	0.245				0.064	0.081				
*Limnodrilus* sp.	CGs									0.210	
*Limnodrilus hoffmeisteri*	CGs		0.010	0.222		0.095	0.052	0.032	0.081		0.042
*Tubifex sinicus*	CGs			0.078							
*Gammarus* sp.	CGs	0.262	0.169	0.329	0.102	0.225	0.024		0.193	0.029	0.048
*Hydropsyche* sp.	CFs						0.139	0.026	0.098	0.103	0.087

Note: CGs = collector-gatherers; PRs = predators; SCs = scrapers; CFs = collector-filterers; SHs = shredders; CPOM = coarse particulate organic matter; FPOM = fine particulate organic matter.

**Table 4:** The spatiotemporal variation in FFG parameters of macroinvertebrates.

Items	Parameters	Gorge Area	Plain Area	March	May	July	October
Material cycling	F1	14.63	45.49	9.06	25.86	42.49	37.22
	F2	2.89	4.51	1.6	3.29	4.76	4.85
	F3	0.08	1.25	0.09	0.52	0.36	1.49
	F4	277.67	89.86	115.39	125.99	407.61	120.20
Longitudinal transport	F5	9.32	33.44	15.81	12.53	19.63	33.18
	F6	0.07	2.08	0.42	0.24	2.68	0.60
Lateral input	F7	4.01	15.38	0.51	12.22	3.08	20.91
	F8	0.03	0.09	0.01	0.08	0.02	0.12
Others	F9	0.03	0.50	0.01	0.14	0.04	0.78
	F10	0.04	0.10	0.04	0.13	0.03	0.06
	F11	0.25	3.39	0.54	0.86	4.12	1.18

**Table 5:** Hilsenhoff Biological Index (*HBI*) and Shannon–Wiener Index (*H*′) of water quality assessment in different months in the upper Yellow River. Among them, the *HBI* is categorized into five levels: <3.9 (Excellent), 3.9–5.4 (Good), 5.4–7.0 (Moderate), 7.0–8.5 (Bad), and >8.5 (Very bad). The *H*′ is divided into five classes: >3.0 (Excellent), 2.0–3.0 (Good), 1.0–2.0 (Moderate), 0–1.0 (Bad), and =0 (Very bad).

Sampling Points		*HBI*				*H*′			
		March	May	July	October	March	May	July	October
Gorge areas	G1	3.03	4.40	2.50	3.20	1.0694	1.5825	0	1.0017
	G2	2.50	3.38	2.51	2.50	0	1.3656	0.0526	0
	G3	7.74	8.26	2.50	8.54	1.7297	1.4095	0	1.8375
	G4	2.82	5.60	6.35	5.70	0.9052	1.9449	1.7210	1.9322
	G5	3.25	4.40	3.66	2.55	1.0409	1.3639	1.1817	0.4912
	G6	8.45	3.47	2.50	3.50	1.8710	1.6627	0	1.2935
	G7	8.44	9.44	9.39	6.00	1.0699	0.7753	0.7993	1.5440
	G8	5.75	8.11	8.76	4.63	1.2826	2.0560	1.2128	1.5774
	G9	9.79	9.22	3.30	4.63	1.2155	1.0934	1.0548	2.2732
	G10	3.98	3.66	2.99	5.97	1.7843	1.7415	0.8390	1.3049
	G11	4.72	8.65	4.44	9.01	0.9757	0.9913	1.8955	0.8343
	G12	4.77	4.81	6.63	8.79	1.7238	1.6669	1.8446	1.5579
	G13	5.46	9.25	6.02	8.77	1.6088	2.0205	2.2824	0.9939
	G14	5.54	5.55	4.94	5.62	1.7500	2.0121	1.8250	1.1596
	G15	2.65	6.09	2.86	3.08	0.2762	1.5810	0.4983	0.9427
	G16	4.47	8.40	4.04	6.38	1.9742	1.6821	2.0169	0.8443
	G17	4.55	5.99	5.21	9.45	2.1051	2.0040	0.9013	0.8315
	G18	5.33	4.89	2.91	5.99	0.7475	2.0223	0.7622	1.8464
	Average value	5.18	6.30	4.53	5.80	1.2849	1.6098	1.0493	1.2370
Plain areas	P1	2.60	7.14	3.43	4.59	0.7026	2.1147	1.1795	1.3303
	P2	4.49	7.95	3.79	6.93	1.4098	2.4468	2.4194	2.2859
	P3	7.77	5.07	6.34	4.63	1.8619	2.1299	2.5318	0.6508
	P4	8.35	7.59	8.87	5.19	1.9356	1.4924	1.3481	2.2788
	P5	8.65	4.78	9.37	7.87	1.0958	2.7962	0.8698	1.9306
	P6	5.09	6.30	2.56	6.91	0.8998	1.7152	0.2996	1.2210
	P7	3.69	7.29	5.02	8.03	1.4210	2.2356	1.7527	1.5016
	P8	7.96	7.63	4.80	5.65	2.1473	1.8724	1.6930	1.7161
	P9	5.04	6.80	5.41	6.34	1.9593	1.8341	1.3965	1.5991
	P10	6.96	7.63	5.02	4.09	1.3796	1.2988	1.2509	1.3101
	P11	6.58	4.44	5.00	5.78	1.8911	1.4098	1.9610	1.4565
	P12	9.29	3.84	7.78	4.71	0.8841	2.4235	2.6426	2.5771
	P13	4.62	5.57	5.08	5.37	1.0995	1.7971	1.9046	2.4911
	P14	5.07	5.63	4.13	4.56	1.1596	2.0230	0.9226	2.3938
	P15	4.09	4.58	4.47	3.92	0.2762	2.4695	1.4864	1.3998
	Average value	6.02	6.15	5.40	5.64	1.3415	2.0039	1.5772	1.7428

## Data Availability

The data presented in this study are available on request from the corresponding author.

## Supplementary Materials

The following supporting information can be downloaded at: https://www.mdpi.com/article/10.3390/biology13100791/s1, Table S1: Latitude and longitude of sampling points in the gorge and plain areas of the upper Yellow River; Table S2: Species composition and functional feeding group division of macroinvertebrates in the upper Yellow River; Table S3: The density of functional feeding groups of macroinvertebrates in different sections of the upper Yellow River in March (ind./m^2^); Table S4: The density of functional feeding groups of macroinvertebrates in different sections of the upper Yellow River in May (ind./m^2^); Table S5: The density of functional feeding groups of macroinvertebrates in different sections of the upper Yellow River in July (ind./m^2^); Table S6: The density of functional feeding groups of macroinvertebrates in different sections of the upper Yellow River in October (ind./m2); Table S7: Comparison of water environmental factors in the gorge and plain areas of the upper Yellow River in different months (Mean ± SD).
