# Supplementary material for: The Spatiotemporal Variation and Ecological Evaluation of Macroinvertebrate Functional Feeding Groups in the Upper Yellow River
Source: Biology (Basel). 2024 Oct 2;13(10):791. doi: 10.3390/biology13100791 (PMC11505124; doi:10.3390/biology13100791)
Supplement: Supplementary file 1 [file biology-13-00791-s001.zip › biology-3157668-supplementary.pdf]

**Table S1.** Latitude and longitude of sampling points in the gorge and plain areas of the upper Yellow River

| Sampling points | East longitude | Northern latitude | Sampling points | East longitude | Northern latitude |
|---|---|---|---|---|---|
| G1 | 101°15′46.8″ | 36°3′57.59″ | P1 | 105°2′34.53″ | 37°26′57.92″ |
| G2 | 101°36′54″ | 36°8′42″ | P2 | 105°18′2.52″ | 37°29′32.64″ |
| G3 | 101°57′57.6″ | 36°1′40.8″ | P3 | 105°32′19.32″ | 37°29′31.2″ |
| G4 | 102°4′15.6″ | 35°51′54″ | P4 | 105°47′26.88″ | 37°33′54″ |
| G5 | 102°29′5.99″ | 35°52′8.4″ | P5 | 105°53′36.96″ | 37°37′37.2″ |
| G6 | 102°50′27.6″ | 35°51′25.2″ | P6 | 105°56′6″ | 37°47′20.4″ |
| G7 | 103°20′49.19″ | 35°55′4.8″ | P7 | 105°58′15.6″ | 37°49′37.2″ |
| G8 | 103°15′54″ | 35°50′38.4″ | P8 | 105°59′13.2″ | 37°52′22.8″ |
| G9 | 103°21′10.79″ | 36°7′15.6″ | P9 | 106°5′9.59″ | 37°57′24.12″ |
| G10 | 103°53′42″ | 36°4′22.8″ | P10 | 106°13′3″ | 38°9′7.19″ |
| G11 | 104°10′1.2″ | 36°19′55.2″ | P11 | 106°19′8.4″ | 38°17′49.2″ |
| G12 | 104°17′20.4″ | 36°21′32.4″ | P12 | 106°32′42″ | 38°37′8.4″ |
| G13 | 104°37′33.6″ | 36°34′15.6″ | P13 | 106°39′10.79″ | 38°48′54″ |
| G14 | 104°41′13.2″ | 36°43′1.19″ | P14 | 106°50′56.4″ | 39°1′19.19″ |
| G15 | 104°35′38.4″ | 36°47′20.4″ | P15 | 106°47′56.4″ | 39°19′8.4″ |
| G16 | 104°21′57.59″ | 36°53′49.19″ | | | |
| G17 | 104°18′39.6″ | 37°9′10.79″ | | | |
| G18 | 104°25′49.8″ | 37°11′42.01″ | | | |

Note: G1-G18 represent the sampling points in the gorge area, and P1-P15 represent the sampling points in the plain area.

**Table S2.** Species composition and functional feeding group division of macroinvertebrates in the upper Yellow River

| Order | Family | Taxonomic unit | Gorge area | Plain area | FFGs |
|---|---|---|---|---|---|
| Ephemeroptera | Baetidae | 1. *Baetis* sp. | + | + | CGs |
| | Heptageniidae | 2. *Eodyonurus* sp. | + | + | SCs |
| | | 3. *Ecdyonurus tobiironis* | | + | SCs |
| Plecoptera | Chloroperlidae | 4. *Sweltsa* sp. | + | + | PRs |
| | Perlidae | 5. *Oyamia* sp. | | + | PRs |
| Diptera | Tipulidae | 6. *Tipula* sp. | + | + | SHs |
| | | 7. *Dicranota* sp. | + | + | SHs |
| | | 8. *Nephrotoma* sp. | + | + | SHs |
| | | 9. *Antocha* sp. | | + | SHs |
| | | 10. *Tipula praepotens* | | + | SHs |
| | Simuliidae | 11. *Simulium aemulum* | + | | CFs |
| | Chironomidae | 12. *Chironomus* sp. | + | + | CGs |
| | | 13. *Chironomus riparius* | + | + | CGs |
| | | 14. *Chironomus anthracinus* | + | + | CGs |
| | | 15. *Chironomus flaviplumus* | + | + | CGs |

| | | | | | |
|---|---|---|---|---|---|
| | | 16. *Chironomus acerbiphilus* | + | | CGs |
| | | 17. *Macropelopia paranebulosa* | + | | CGs |
| | | 18. *Polypedilum* sp. | + | + | CGs |
| | | 19. *Hamischia fuscimana* | + | | CGs |
| | | 20. *Cricotopu triannulatus* | + | | CGs |
| | | 21. *Cryptochironomus* sp. | | + | PRs |
| | Dolichopodidae | 22. *Rhaphium* sp. | + | + | PRs |
| Cryptocerata | Corixidae | 23. *Micronecta grisea* | + | + | PRs |
| | | 24. *Sigare sbustriata* | + | + | PRs |
| | Aphelocheiridae | 25. *Aphelocheirus nawae* | + | + | PRs |
| | Naucoridae | 26. *Llyocoris* sp. | | + | PRs |
| Trichoptera | Hydopsychidae | 27. *Hydropsyche* sp. | + | + | CFs |
| | | 28. *Cheumatopsyche brevilineata* | + | + | CFs |
| | Limnephilidae | 29. *Limnephilus* sp. | + | + | SHs |
| | Rhyacophilidae | 30. *Rhyacophila* sp. | | + | PRs |
| Odonata | Gomphidae | 31. *Davidius* sp. | | + | PRs |
| | | 32. *Anisogomphus maacki* | | + | PRs |
| | Libellulidae | 33. *Deielia phaon* | | + | PRs |
| | Aeshnidae | 34. *Anax nigrofasciatus nigrofasciatus* | + | + | PRs |
| | Coenagriidae | 35. *Ischnura labata* | + | + | PRs |
| | Calopterygidae | 36. *Calopteryx atratum* | | + | PRs |
| Coleoptera | Chrysomelidae | 37. *Galerucella* sp. | | + | PRs |
| | Psephenidae | 38. *Psephenoides* sp. | + | + | SCs |
| | Dytiscidae | 39. *Dytiscus* sp. | | + | PRs |
| | | 40. *Oreodytes* sp. | | + | PRs |
| | | 41. *Nebrioporus hostilis* | | + | PRs |
| | Curculionidae | 42. *Echinocnemus* sp. | | + | SHs |
| Amphipoda | Gammaridae | 43. *Gammarus* sp. | + | + | CGs |
| | Palaemonidae | 44. *Palaemon modestus* | + | + | SHs |
| | | 45. *Palaemonetes sinensis* | | + | SHs |
| Pharyngobdellida | Herpobdellidae | 46. *Herpobdella octoculata* | + | + | PRs |
| Rhynchobdellida | Glossiphoniidae | 47. *Helobdella stagnalis* | + | | PRs |
| | | 48. *Glossiphonia lata* | + | + | PRs |
| Oligochaeta plesiopora | Tubificidae | 49. *Limnodrilus* sp. | + | + | CGs |
| | | 50. *Limnodrilus hoffmeisteri* | + | + | CGs |
| | | 51. *Branchiura sowerbyi* | + | + | CGs |
| | | 52. *Tubifex tubifex* | + | + | CGs |
| | | 53. *Tubifex sinicus* | + | + | CGs |
| Basommatophora | Lymnaeidae | 54. *Radix auricularia* | + | + | SCs |
| | | 55. *Radix ovata* | + | + | SCs |
| | | 56. *Galba truncatula* | | + | SCs |

| | | | | | |
|---|---|---|---|---|---|
| | Hydrobiidae | 57. *Parafossarulus* sp. | + | + | SCs |
| | | 58. *Parafossarulus striatulus* | + | | SCs |
| | Planorbidae | 59. *Gyraulus albus* | | + | SCs |
| | | 60. *Gyraulus convexiusculus* | | + | SCs |
| | Bithyniidae | 61. *Bithynia fuchsiana* | | + | SCs |
| | Viviparidae | 62. *Cipangopaludma chinensis* | | + | SCs |
| | | 63. *Bellamya purificata* | | + | SCs |
| Unionoida | Unionidae | 64. *Anodonta woodiana* | | + | CFs |
| Tricladida | Dugesiidae | 65. *Dugesiidae japonica* | + | | PRs |

Note: CGs = collector-gatherers, PRs = predators, SCs = scrapers, CFs = collector-filterers, SHs = shredders, and "+" indicates that the species has been collected in this river section. The same as below.

**Table S3.** The density of functional feeding groups of macroinvertebrates in different sections of the upper Yellow River in March (ind./$m^2$).

| Sampling points | | CFs | CGs | PRs | SCs | SHs |
|---|---|---|---|---|---|---|
| Gorge areas | G1 | 0 | 116.67 | 0 | 0 | 0 |
| | G2 | 0 | 50.00 | 0 | 0 | 0 |
| | G3 | 0 | 223.33 | 0 | 0 | 0 |
| | G4 | 0 | 176.67 | 0 | 0 | 0 |
| | G5 | 0 | 40.00 | 0 | 0 | 0 |
| | G6 | 10.00 | 46.67 | 10.00 | 0 | 0 |
| | G7 | 0 | 226.67 | 0 | 6.67 | 0 |
| | G8 | 0 | 282.22 | 20.00 | 2.22 | 0 |
| | G9 | 0 | 91.11 | 0 | 0 | 0 |
| | G10 | 20.00 | 100.00 | 0 | 6.67 | 0 |
| | G11 | 0 | 157.78 | 0 | 0 | 0 |
| | G12 | 5.00 | 183.33 | 0 | 33.33 | 0 |
| | G13 | 0 | 142.22 | 0 | 0 | 0 |
| | G14 | 2.22 | 260.00 | 0 | 0 | 0 |
| | G15 | 0 | 46.67 | 0 | 0 | 0 |
| | G16 | 102.22 | 306.67 | 0 | 44.44 | 0 |
| | G17 | 22.22 | 148.89 | 0 | 55.56 | 0 |
| | G18 | 0 | 386.67 | 2.22 | 0 | 0 |
| Plain areas | P1 | 13.33 | 150.00 | 10.00 | 0 | 0 |
| | P2 | 103.33 | 23.33 | 0 | 33.33 | 0 |
| | P3 | 0 | 203.33 | 0 | 0 | 0 |
| | P4 | 0 | 126.67 | 22.22 | 0 | 0 |
| | P5 | 0 | 44.44 | 4.44 | 0 | 0 |
| | P6 | 60.00 | 15.56 | 2.22 | 0 | 0 |
| | P7 | 53.33 | 0 | 10.00 | 0 | 13.33 |
| | P8 | 0 | 43.33 | 10.00 | 6.67 | 0 |
| | P9 | 0 | 26.67 | 16.67 | 20.00 | 0 |

| | | | | | | |
|---|---|---|---|---|---|---|
| | P10 | 3.33 | 66.67 | 0 | 3.33 | 0 |
| | P11 | 6.67 | 10.00 | 0 | 10.00 | 3.33 |
| | P12 | 0 | 95.56 | 0 | 0 | 0 |
| | P13 | 53.33 | 0 | 0 | 26.67 | 0 |
| | P14 | 0 | 0 | 10.00 | 46.67 | 0 |
| | P15 | 66.67 | 0 | 0 | 3.33 | 0 |

**Table S4.** The density of functional feeding groups of macroinvertebrates in different sections of the upper Yellow River in May (ind./$m^2$).

| Sampling points | | CFs | CGs | PRs | SCs | SHs |
|---|---|---|---|---|---|---|
| Gorge areas | G1 | 0 | 256.67 | 0 | 0 | 0 |
| | G2 | 23.33 | 146.67 | 0 | 16.67 | 0 |
| | G3 | 0 | 170.00 | 0 | 23.33 | 6.67 |
| | G4 | 0 | 236.67 | 16.67 | 0 | 0 |
| | G5 | 0 | 170.00 | 0 | 23.33 | 6.67 |
| | G6 | 126.67 | 80.00 | 0 | 0 | 0 |
| | G7 | 0 | 513.33 | 0 | 0 | 0 |
| | G8 | 22.22 | 71.11 | 22.22 | 0 | 0 |
| | G9 | 17.78 | 340.00 | 2.22 | 0 | 0 |
| | G10 | 0 | 106.67 | 0 | 20.00 | 16.67 |
| | G11 | 0 | 20.00 | 66.67 | 0 | 0 |
| | G12 | 0 | 96.67 | 0 | 13.33 | 0 |
| | G13 | 0 | 153.33 | 23.33 | 0 | 0 |
| | G14 | 0 | 173.33 | 0 | 30.00 | 0 |
| | G15 | 0 | 63.33 | 0 | 0 | 0 |
| | G16 | 0 | 60.00 | 46.67 | 0 | 0 |
| | G17 | 0 | 76.67 | 0 | 53.33 | 0 |
| | G18 | 16.67 | 33.33 | 23.33 | 66.67 | 0 |
| Plain areas | P1 | 0 | 43.33 | 13.33 | 20.00 | 13.33 |
| | P2 | 0 | 153.33 | 53.33 | 0 | 0 |
| | P3 | 50.00 | 10.00 | 36.67 | 16.67 | 26.67 |
| | P4 | 0 | 70.00 | 10.00 | 73.33 | 0 |
| | P5 | 0 | 113.33 | 53.33 | 46.67 | 23.33 |
| | P6 | 0 | 33.33 | 56.67 | 83.33 | 0 |
| | P7 | 16.67 | 166.67 | 0 | 53.33 | 0 |
| | P8 | 0 | 145.00 | 95.00 | 85.00 | 0 |
| | P9 | 50.00 | 90.00 | 125.00 | 0 | 0 |
| | P10 | 0 | 75.00 | 0 | 0 | 45.00 |
| | P11 | 0 | 0 | 0 | 45.00 | 95.00 |
| | P12 | 50.00 | 0 | 33.33 | 63.33 | 16.67 |
| | P13 | 0 | 10.00 | 40.00 | 63.33 | 0 |
| | P14 | 0 | 63.33 | 0 | 30.00 | 73.33 |
| | P15 | 40.00 | 13.33 | 0 | 26.67 | 80.00 |

**Table S5.** The density of functional feeding groups of macroinvertebrates in different sections of the upper Yellow River in July (ind./m$^2$).

| Sampling points | | CFs | CGs | PRs | SCs | SHs |
|---|---|---|---|---|---|---|
| Gorge areas | G1 | 0 | 323.33 | 0 | 0 | 0 |
| | G2 | 0 | 556.67 | 3.33 | 0 | 0 |
| | G3 | 0 | 40.00 | 0 | 0 | 0 |
| | G4 | 0 | 233.33 | 0 | 3.33 | 0 |
| | G5 | 11.11 | 268.89 | 0 | 0 | 0 |
| | G6 | 0 | 390.00 | 0 | 0 | 0 |
| | G7 | 0 | 4303.33 | 0 | 0 | 23.33 |
| | G8 | 0 | 683.33 | 0 | 10.00 | 10.00 |
| | G9 | 0 | 233.33 | 10.00 | 36.67 | 0 |
| | G10 | 0 | 302.22 | 2.22 | 22.22 | 2.22 |
| | G11 | 26.67 | 313.33 | 6.67 | 6.67 | 6.67 |
| | G12 | 16.67 | 1136.67 | 10.00 | 16.67 | 0 |
| | G13 | 0 | 162.22 | 6.67 | 40.00 | 0 |
| | G14 | 0 | 1056.67 | 0 | 53.33 | 0 |
| | G15 | 0 | 358.00 | 0 | 44.00 | 0 |
| | G16 | 86.67 | 500.00 | 3.33 | 190.00 | 0 |
| | G17 | 6.67 | 476.67 | 0 | 10.00 | 6.67 |
| | G18 | 0 | 220.00 | 3.33 | 26.67 | 0 |
| Plain areas | P1 | 13.33 | 203.33 | 0 | 76.67 | 0 |
| | P2 | 10.00 | 3.33 | 6.67 | 0 | 10.00 |
| | P3 | 0 | 110.00 | 3.33 | 10.00 | 0 |
| | P4 | 0 | 186.67 | 0 | 3.33 | 13.33 |
| | P5 | 0 | 466.67 | 0 | 0 | 0 |
| | P6 | 8.00 | 262.00 | 0 | 4.00 | 0 |
| | P7 | 0 | 23.33 | 6.67 | 0 | 0 |
| | P8 | 3.33 | 350.00 | 0 | 66.67 | 3.33 |
| | P9 | 28.57 | 22.86 | 17.14 | 248.57 | 2.86 |
| | P10 | 70.00 | 6.67 | 0 | 113.33 | 3.33 |
| | P11 | 50.00 | 86.67 | 23.33 | 280.00 | 0 |
| | P12 | 0 | 56.67 | 3.33 | 10.00 | 6.67 |
| | P13 | 66.67 | 10.00 | 3.33 | 70.00 | 10.00 |
| | P14 | 110.00 | 3.33 | 10.00 | 10.00 | 0 |
| | P15 | 140.00 | 0 | 6.67 | 50.00 | 3.33 |

**Table S6.** The density of functional feeding groups of macroinvertebrates in different sections of the upper Yellow River in October (ind./m$^2$).

| Sampling points | | CFs | CGs | PRs | SCs | SHs |
|---|---|---|---|---|---|---|
| Gorge areas | G1 | 0 | 210.00 | 0 | 0 | 0 |
| | G2 | 0 | 140.00 | 0 | 0 | 0 |

| | | | | | | |
|---|---|---|---|---|---|---|
| | G3 | 0 | 75.00 | 0 | 20.00 | 10.00 |
| | G4 | 0 | 185.00 | 0 | 35.00 | 0 |
| | G5 | 0 | 166.67 | 0 | 0 | 20.00 |
| | G6 | 20.00 | 295.00 | 0 | 0 | 0 |
| | G7 | 0 | 56.67 | 0 | 0 | 16.67 |
| | G8 | 95.00 | 220.00 | 0 | 0 | 0 |
| | G9 | 40.00 | 63.33 | 3.33 | 13.33 | 23.33 |
| | G10 | 0 | 185.00 | 0 | 15.00 | 0 |
| | G11 | 0 | 70.00 | 0 | 0 | 15.00 |
| | G12 | 0 | 95.00 | 0 | 35.00 | 0 |
| | G13 | 0 | 170.00 | 0 | 0 | 50.00 |
| | G14 | 0 | 0 | 0 | 60.00 | 25.00 |
| | G15 | 0 | 80.00 | 0 | 0 | 45.00 |
| | G16 | 0 | 275.00 | 0 | 0 | 0 |
| | G17 | 0 | 105.00 | 5.00 | 0 | 0 |
| | G18 | 0 | 0 | 25.00 | 20.00 | 5.00 |
| Plain areas | P1 | 0 | 85.00 | 5.00 | 150.00 | 15.00 |
| | P2 | 35.00 | 230.00 | 5.00 | 145.00 | 10.00 |
| | P3 | 335.00 | 40.00 | 10.00 | 0 | 0 |
| | P4 | 0 | 45.00 | 5.00 | 140.00 | 40.00 |
| | P5 | 20.00 | 20.00 | 105.00 | 15.00 | 0 |
| | P6 | 0 | 50.00 | 5.00 | 0 | 30.00 |
| | P7 | 0 | 115.00 | 0 | 0 | 5.00 |
| | P8 | 120.00 | 80.00 | 0 | 0 | 5.00 |
| | P9 | 0 | 45.00 | 0 | 85.00 | 15.00 |
| | P10 | 175.00 | 0 | 10.00 | 30.00 | 35.00 |
| | P11 | 0 | 140.00 | 0 | 0 | 0 |
| | P12 | 0 | 10.00 | 25.00 | 125.00 | 85.00 |
| | P13 | 0 | 10.00 | 95.00 | 230.00 | 85.00 |
| | P14 | 0 | 15.00 | 35.00 | 70.00 | 65.00 |
| | P15 | 255.00 | 0 | 0 | 40.00 | 90.00 |

**Table S7.** Comparison of water environmental factors in the gorge and plain areas of the upper Yellow River in different months (Mean ± SD)

| Environmental factors | March | | May | | July | | October | |
|---|---|---|---|---|---|---|---|---|
| | Gorge area | Plain area | Gorge area | Plain area | Gorge area | Plain area | Gorge area | Plain area |
| T (°C) | 5.03±1.57 | 6.29±1.21 | 13.86±2.73 | 16.31±0.96 | 16.14±4.32 | 23.71±2.08 | 13.63±1.24 | 18.11±1.77 |
| pH | 8.04±0.54 | 8.72±0.18 | 8.49±0.14 | 8.46±0.09 | 8.53±0.26 | 7.93±0.25 | 8.84±0.12 | 8.16±0.12 |
| Cond (μS/cm) | 386.31±90.36 | 957.33±94.57 | 431.92±123.35 | 610.27±78.07 | 798.32±393.48 | 1078.93±280.10 | 441.87±101.64 | 619.47±108.63 |
| DO (mg/L) | 11.99±1.38 | 10.76±0.82 | 7.85±1.07 | 7.45±0.45 | 8.03±1.23 | 7.78±0.64 | 8.65±0.88 | 7.05±0.65 |
| $PO_4^{3-}$ (mg/L) | 0.04±0.01 | 0.05±0.01 | 0.01±0.00 | 0.02±0.00 | 0.03±0.05 | 0.14±0.06 | 0.05±0.02 | 0.05±0.01 |

| | | | | | | | | |
|---|---|---|---|---|---|---|---|---|
| TP (mg/L) | 0.07±0.03 | 0.06±0.01 | 0.08±0.11 | 0.10±0.07 | 0.09±0.07 | 0.13±0.09 | 0.04±0.02 | 0.05±0.01 |
| TN (mg/L) | 0.89±0.09 | 0.97±0.08 | 0.21±0.11 | 0.29±0.05 | 1.40±0.59 | 2.42±0.90 | 0.95±0.04 | 0.89±0.16 |
| $NH_3$-N (mg/L) | 0.33±0.23 | 0.50±0.14 | 0.26±0.06 | 0.33±0.06 | 0.11±0.09 | 0.14±0.12 | 0.31±0.10 | 0.34±0.07 |
| COD (mg/L) | 2.11±0.39 | 2.94±0.62 | 2.31±1.09 | 2.39±1.25 | 2.42±0.78 | 3.23±0.73 | 2.21±0.97 | 2.09±1.25 |
| Chl-a (μg/L) | 1.78±1.20 | 1.30±1.90 | 1.50±1.57 | 1.02±0.56 | 0.29±0.09 | 0.35±0.12 | 0.83±0.69 | 1.24±1.81 |
| SSol (mg/L) | 5.06±3.78 | 4.73±3.21 | 3.89±2.77 | 5.91±7.68 | 13.00±6.00 | 28.33±16.26 | 28.78±10.34 | 32.07±8.78 |
| SSed (mg/L) | 18.44±12.49 | 109.00±107.36 | 143.22±290.20 | 564.47±957.54 | 3477.94±1087.13 | 5731.33±2431.79 | 2706.50±2761.68 | 4586.13±3536.90 |

Note: T = water temperature, DO = dissolved oxygen, Cond = conductivity, $PO_4^{3-}$ = orthophosphate, TP = total phosphorus, TN = total nitrogen, $NH_3$-N = ammonia nitrogen, CODMn = chemical oxygen demand, Chl-a = chlorophyll-a, SSol = suspended solids, and SSed = suspended sediment.
